# The Emerging Role of the Histone H2AK13/15 Ubiquitination: Mechanisms of Writing, Reading, and Erasing in DNA Damage Repair and Disease

**DOI:** 10.3390/cells14040307

**Published:** 2025-02-18

**Authors:** Qi Shu, Yun Liu, Huasong Ai

**Affiliations:** School of Pharmaceutical Sciences, Shanghai Frontiers Science Center of Drug Target Identification and Delivery, Shanghai Jiao Tong University, Shanghai 200240, China

**Keywords:** DNA damage repair (DDR), histone ubiquitination, H2AK13/15ub, RNF168, 53BP1, BARD1, USP3, structure, human diseases

## Abstract

Histone modifications serve as molecular switches controlling critical cellular processes. The ubiquitination of histone H2A at lysines 13 and 15 (H2AK13/15ub) is a crucial epigenetic modification that coordinates DNA repair and genome stability during the DNA damage response (DDR). This epigenetic mark is dynamically regulated by three functional protein groups: “writer” enzymes (e.g., E3 ubiquitin ligase RNF168 that catalyzes H2AK13/15ub formation), “reader” proteins (including 53BP1 and BRCA1-BARD1 that recognize the mark to guide DNA repair), and “eraser” deubiquitinases (such as USP3 and USP16 that remove the modification). Dysregulation of the precisely coordinated network of H2AK13/15ub is strongly associated with various diseases, including RIDDLE syndrome, neurodegenerative disorders, immune deficiencies, and breast cancer. This review systematically analyzes the dynamic regulation of H2AK13/15ub in DDR and explores its therapeutic potential for disease intervention.

## 1. Introduction

In eukaryotic organisms, chromatin functions as a sophisticated assembly of DNA and proteins essential for genome organization [1]. The nucleosome core particle (NCP), which is the basic structural unit of chromatin, is formed by approximately 147 bp of superhelical DNA wrapped around a histone octamer, consisting of two H2A/H2B heterodimers and an H3/H4 heterotetramer [1]. Histones are modified with a diverse and complex range of post-translational modifications (PTMs), including methylation, acetylation, phosphorylation, ubiquitination, and ubiquitin-like modifications such as SUMO and UFM [2]. These PTMs are pivotal in either altering the chromatin structure or recruiting effector proteins, regulating essential DNA-related processes, including DNA replication, transcriptional activation, gene silencing, and DNA damage repair [2,3,4,5]. The complexity and diversity of histone PTMs and their spatial and temporal combinations play crucial roles as epigenetic marks, collectively encapsulating the essence of the “histone code” hypothesis [6].

Unlike small-molecule modifications like methylation (14 Da) and acetylation (42 Da), histone ubiquitination involves the attachment of the significantly larger ubiquitin moiety (~8.5 kDa) to substrate lysine residues, which facilitates additional protein interactions and confers unique biological functions. Histone ubiquitination occurs predominantly on the H2A and H2B, with H2AK119 ubiquitination being essential for transcriptional silencing [7,8,9] and H2AK13/15 ubiquitination playing a key role in DNA damage repair [10]. H2BK120 ubiquitination regulates transcriptional activation by modulating chromatin fiber density and facilitating the recruitment of downstream proteins such as the DOT1 Like histone lysine methyltransferase (Dot1L) [11,12] and Complex of Proteins Associated with Set1 (COMPASS) complex [13,14]. Additionally, other ubiquitination sites in H2A and H2B, such as H2AK125/127/129 [15] and H2BK34 [16,17,18], are related to DNA damage repair, chromatin remodeling, and transcriptional regulation, thus contributing to a complex and dynamic epigenetic network.

Recently, the H2AK13/15 ubiquitination has garnered increasing attention regarding its critical role in DNA damage repair [10,19,20,21,22]. Dysregulation of H2AK13/15 ubiquitination has been shown to impair DNA repair efficiency and lead to inappropriate repair pathway selection, which is associated with various diseases, including neurological disorders [23], immune system diseases [24], and breast cancer [25]. The dynamic regulation of H2AK13/15 ubiquitination operates through a dynamic “write, read, erase” ubiquitin code that is critical for maintaining genomic stability and managing pathology. The E3 ubiquitin ligase RNF168 operates as a dedicated “writer”, catalyzing H2AK13/15 ubiquitination deposition in a nucleosomal context [10]. The resulting H2AK13/15ub creates a signaling platform that recruits DNA damage repair factors, which act as “readers” to determine the repair pathway choice of non-homologous end joining (NHEJ) or homologous recombination (HR), notably involving the 53BP1 and BRCA1-BARD1 complex [21,22]. Concurrently, several deubiquitinating enzymes (e.g., USP3 [26], USP16 [27], USP44 [28], USP51 [29], and POH1 [30,31]) have been reported to function as “erasers” to remove the ubiquitin modifications from H2A to maintain the dynamic balance of H2AK13/15ub signaling. In this review, we provide a comprehensive overview of the molecular mechanisms governing the writing, reading, and erasing of H2A K13/15 ubiquitination and discuss its implications in disease development.

## 2. Writing: RNF168-Mediated H2AK13/15ub

### 2.1. Discovery, Function, and Domain Composition of RNF168

**Figure 1:**
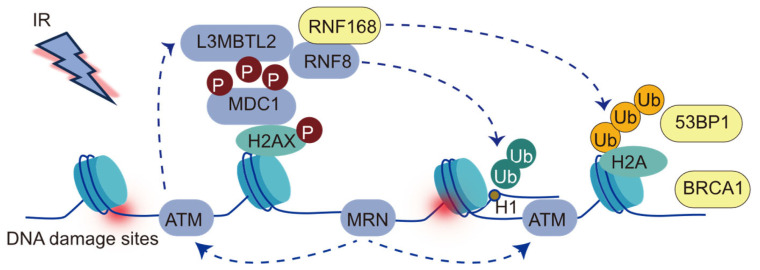
The cascade of events in the DNA damage response to double-strand breaks (DSBs). DSB induction, represented by a lightning bolt, leads to the phosphorylation of H2AX, resulting in γH2AX formation by ATM kinase. γH2AX recruits MDC1, which in turn recruits RNF8. RNF8-mediated ubiquitination of H1 or L3MBTL2 has been proposed to recruit RNF168. RNF168 mediates K63-linked (orange) ubiquitination on lysines 13 and 15 of H2A-type histones. H2AK13/15 ubiquitination serves as a recruitment platform for downstream mediators of the DSB response, including the BRCA1-A complex and 53BP1.

RNF168, characterized in 2009 as a chromatin-associated protein, contains ubiquitin-binding domains and a RING domain [32]. It functions as an E3 ubiquitin ligase, specifically mediating histone H2A lysine 13 and 15 ubiquitination at sites of DNA damage [32]. Upon the occurrence of DNA double-strand breaks (DSBs), the damage site is initially recognized by the MRE11-RAD50-NBS1 (MRN) complex, a key component of the DNA damage response network (Figure 1) [33]. The MRN complex subsequently recruits ATM kinase, which phosphorylates histone H2AX at Ser139, forming γH2AX [10,33]. RNF8 then catalyzes the formation of K63-linked ubiquitin chains on substrates such as L3MBTL2 or linker histone H1, facilitating the recruitment of RNF168 to the DNA damage sites [34,35]. Once localized, RNF168 ubiquitinates histone H2A at the N-terminal lysine residues K13/15 in the nucleosomal context [36], generating binding sites that recruit downstream DNA repair factors, including 53BP1 and BRCA1/BARD1 [37,38].

**Figure 2:**
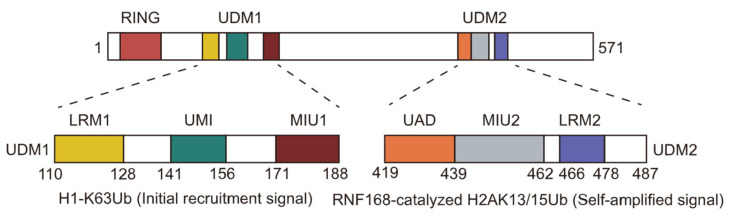
Schematic representation of RNF168 functional domain. The RING domain, the E3 ligase catalytic domain, was responsible for the ubiquitination of H2AK13/15. The UDM1 domain plays a crucial role in the initial recruitment of RNF168 to DNA damage sites by binding to polyubiquitinated H1.0. Additionally, the UDM2 domain interacts with the H2AK13/15 ubiquitinated nucleosome, facilitating the amplification of RNF168 signaling.

The full-length human RNF168 protein is comprised of 571 amino acids and features an N-terminal RING structural domain (residues 15–58) along with two ubiquitin-binding domains: UDM1 (ubiquitin-dependent DSB recruitment module 1, residues 110–188) and UDM2 (ubiquitin-dependent DSB recruitment module 2, residues 419–487) (Figure 2) [32]. The RING domain serves as the catalytic core responsible for the E3 enzyme activity of RNF168 and binds to the nucleosome H2A-H2B acidic patch [39]. Both UDM1 and UDM2 contain several ubiquitin-binding motifs: UDM1 contains LRM, UMI, and MIU1 motifs, while UDM2 contains MIU2 and LRM2 motifs. UDM1 has been reported to bind the K63-linked ubiquitin chain on histone H1, playing a role in the initial recruitment of RNF168 to DSB sites [34]. Additionally, UDM2 recognizes the ubiquitinated product of RNF168, specifically H2AK13/15ub, and promotes the amplification of ubiquitination signals at DSB sites [37,38,39,40].

RNF168 plays a pivotal role in modulating the effectiveness of anticancer drugs through its ubiquitination-related regulatory mechanisms [41,42,43]. RNF168 influences the sensitivity of cancer cells to specific chemotherapeutic agents by promoting distinct forms of ubiquitination, resulting in the degradation of proteins and impacting how cells respond to treatment [44,45,46]. For example, in breast cancer, RNF168 has been demonstrated to promote K48-linked ubiquitination of FOXM1, targeting it for proteasomal degradation, consequently enhancing cancer cell sensitivity to doxorubicin-class chemotherapeutic agents [44]. Furthermore, RNF168 directly interacts with topoisomerase IIα (TOP2α) and facilitates the specific K63-linked polyubiquitination [45]. Notably, the absence of RNF168 renders cells intrinsically resistant to TOP2α catalytic inhibitors, such as ICRF-193, and cytotoxic anticancer agents like etoposide (VP-16) [46]. Dysfunction of RNF168 has been closely linked to a variety of human diseases, including breast cancer [41], esophageal cancer [42,43], and RIDDLE syndrome—a rare immunodeficiency and radiation sensitivity disorder associated with defects in DSB repair [47].

### 2.2. RNF168 Specifically Ubiquitinates Nucleosomal H2AK13/15 Sites

**Figure 3:**
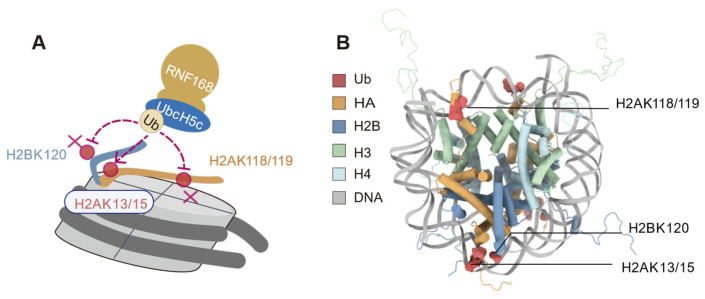
Selective ubiquitination of histone H2A by RNF168. (**A**) Schematic representation of RNF168-mediated ubiquitination at K13/K15 on histone H2A. No significant ubiquitination is observed at lysines 118 and 119 (K118/K119) of H2A or lysine 120 (K120) of H2B, as indicated by the cross symbol (**B**) Structural model showing the nucleosome core particle, highlighting the targeted lysine residues (H2AK13/15, H2AK118/119, and H2BK120) and their spatial positioning relative to the DNA and histone octamer. (Figure created with Chimerax 1.9).

The monomeric E3 ubiquitin ligase RNF168 exhibits precise ubiquitination site selectivity when acting on nucleosome substrates. In vitro ubiquitination assays revealed that RNF168 exhibits minimal ubiquitination activity on nucleosomes with H2A K13R/K15R mutations while retaining the ability to ubiquitinate nucleosomes with H2A K118Q/K119Q mutations (Figure 3) [10]. Furthermore, no significant bands were observed with the H2BK120ub antibody, indicating that RNF168 does not ubiquitinate the H2BK120 site [10]. These results indicate that RNF168 preferentially ubiquitinates the K13 and K15 sites of nucleosome H2A in vitro [10,19]. In H2AX-deficient mouse embryonic fibroblasts (MEFs), the H2AK13/15ub was abolished upon RNF168 depletion, implicating RNF168 as the E3 ligase responsible for H2AX K13/15 ubiquitination in vivo [10].

**Figure 4:**
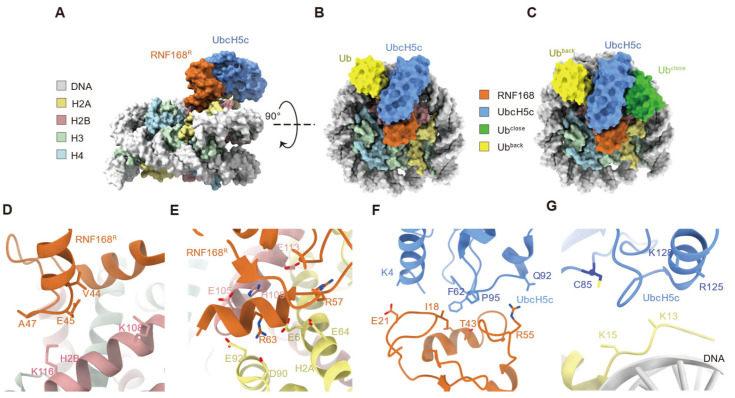
Structural model of the RNF168-UbcH5c-ubiquitin complex on the nucleosome. (**A**,**B**) RNF168 (orange) interacts with the nucleosome core particle via histone H2A (yellow) and DNA (gray). UbcH5c (blue) facilitates ubiquitin (Ub, yellow) transfer. Two orientations of the complex are shown (90° rotation). (**C**) The picture illustrates the spatial positioning of ubiquitin in its “close” (Ub close, green) and “back” (Ub back, yellow) conformations relative to the nucleosome. (**D**) Close-up view of the salt bridge formation between RNF168 and the acidic patch of H2B, along with ion-dipole interactions. (**E**) Close-up view of salt bridge formation between RNF168 and H2A, along with additional interactions at the interface. (**F**) Close-up view of hydrophobic and polar interactions between the RNF168 RING domain and UbcH5c. (**G**) Close-up view of UbcH5c and its α-helix positioned above the SHL 4.5 region of nucleosomal DNA.

To further elucidate the molecular mechanism of RNF168-mediated H2AK13/15 ubiquitination, our group and Hu et al. captured the RNF168-UbcH5c~Ub-nucleosome complex using chemical cross-linking strategies and resolved the cryo-electron microscopy (cryo-EM) structures (Figure 4A–C) [19,39]. In the structures, RNF168 interacts with nucleosomes by positioning a basic helix (residues 59–68) on the acidic patch of H2A and H2B, with the burial of approximately 180 Å^2^ of the surface area that is normally accessible to solvents [19,39]. In the interface, residues V44, E45, and A47 of RNF168 α-helix 1 form salt bridges with H2B K108 and K116 via ion-dipole interactions of the helical cap (Figure 4D) [39]. Additionally, the guanidinium group of RNF168 residue R57 forms a salt bridge with H2A residue E64 [19,39]. In contrast, R63, located in α-helix 2 of RNF168, interacts with H2A residues E61, D90, and E92 (Figure 4E) [19,39]. These interactions between the RNF168 RING domain and nucleosomal surfaces stabilize the conformation of the RNF168 E3 ligase RING domain on the nucleosome [19,39].

RNF168 forms a classical E3-E2 interface with UbcH5c through its RING domain, which is characterized by two Zn^2+^-binding loops (residues 16–22 and 51–55) and α-helix 1 of RNF168, alongside two loops of UbcH5c (residues 56–64 and 91–97) [19,39]. The interactions between RNF168 and UbcH5c are stabilized by a network of hydrophobic contacts (I18, S42, and T43 of RNF168 with A96, F62, and P95 of UbcH5c, respectively) and polar interactions (E21, R55, and S94 of RNF168 with K4, Q92, and P52 of UbcH5c, respectively) (Figure 4F) [19,39]. Additionally, residues R125 and K128 of UbcH5c were positioned above the superhelical loop (SHL) 4.5 region of nucleosomal DNA (Figure 4G) [19]. Through the conserved E3-E2 interface, RNF168 positions UbcH5c toward the N-terminal tail of H2A within the nucleosome, adopting a pre-reaction conformation that facilitates ubiquitin transfer to H2A K13 and K15 [39]. The α-carbon of the H2AK15 lysine is located approximately 10 Å from the catalytic cysteine (C85) of UbcH5c, establishing an optimal arrangement for ubiquitin transfer [39].

Focusing on the ubiquitin moiety, cryo-EM structures of RNF168 for nucleosomal H2AK13/15 monoubiquitination revealed two distinct conformations of ubiquitin [19]. One Ub adopts a conventional closed conformation between the E3 and E2, while the other Ub motif assumes a unique position between UbcH5c and the DNA SHL 3.5 (Figure 4C) [19]. The spatially separated arrangement of the two Ub molecules provided a structural explanation for why the pre-existing Ub does not hinder the second ubiquitination event, given that RNF168 can mediate the dual-monoubiquitination of H2AK13/K15 [19]. Notably, this represents the first structural capture of proximal dual-ubiquitination on the substrate mediated by E3 ligases [19].

The H2AK13/15 ubiquitination is regulated by PTMs of RNF168 and UbcH5c. Phosphorylation of RNF168 at Ser60 inhibits its E3 ligase activity and accelerates its proteolysis, leading to the accumulation of unrepaired DNA and ultimately contributing to genome instability [48]. The S60 residue is located within the basic helix of RNF168, where it interacts stably with H2A residues E61 and E64, positioning RNF168 in the H2A-H2B acidic patch [19]. Phosphorylation at the RNF168 S60 residue introduces a negative charge, creating repulsive forces that disrupt the interaction with the acidic patch of H2A-H2B and weaken the binding of RNF168 [19]. Additionally, UbcH5c K144 ubiquitination facilitates RNF168-dependent H2A ubiquitination [39]. Cryo-EM analysis revealed that the covalent attachment of ubiquitin to UbcH5c at K144 strengthens the E2 backside interaction, potentially promoting the Ub discharge from the E2~Ub to substrate lysine [39]. Unlike the requirement of excess free ubiquitin (Kd~300 μM) for backside binding, the covalent attachment of ubiquitin to UbcH5c ensures stable 1:1 ubiquitin binding to UbcH5c, which would more efficiently facilitate RNF168-mediated H2A ubiquitination [39].

**Figure 5:**
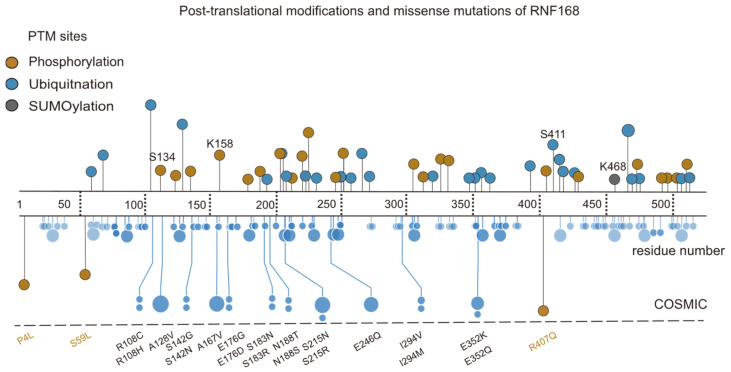
PTMs and missense mutations of RNF168. This schematic aligns the domains of the RNF168 protein (residues 1–571) with PTMs and mutation data. The upper section shows the types and locations of RNF168 PTMs, including phosphorylation (yellow), ubiquitination (blue), and SUMOylation (orange). K468 and S411 are key ubiquitination sites, while S134 and K158 are phosphorylation sites associated with the functional regulation of RNF168. The lower section highlights missense mutations related to RNF168, with specific emphasis on S59L, P4L, and R407Q, which are closely associated with functional loss or abnormal activation of RNF168. Data sources: St. Jude ProteinPaint (https://proteinpaint.stjude.org/, accessed on 1 January 2025) and PhosphoSitePlus (https://www.phosphosite.org/homeAction.action, accessed on 1 January 2025).

Additionally, other PTMs of RNF168 have been identified, including phosphorylation at the Ser134 [49], K158 [50], and Ser411 [51] sites, and SUMOylation at the K468 [52] site. Notably, alterations in the phosphorylation of Lys158 and SUMOylation at Lys468 have been observed in esophageal squamous cell carcinoma and glioblastoma specimens, correlating with poor patient prognosis [50,52]. Furthermore, missense mutations, such as R407T [53], P4L [54], and S59L [55], may impact the functionality of RNF168 (Figure 5), linking these PTMs and mutations to cancer progression. For instance, oral squamous cell carcinoma patients carrying the R407T mutation exhibit impaired DNA damage repair capacity in tumor tissues. These results suggest that the post-translational modifications and mutations of RNF168 could serve as prognostic biomarkers and therapeutic targets in cancer.

Notably, H2A K13/15 ubiquitination has been observed in vivo as polyubiquitin modifications consisting of K27- or K63-linked ubiquitin chains [47,56]. However, in vitro ubiquitination assays using both full-length and truncated RNF168 constructs only produce shorter ubiquitin chains [10,19,20,57,58]. The discrepancy between the in vitro and in vivo ubiquitination outcomes suggests that additional regulators, such as the K63 chain-specific E2 conjugating enzyme UBC13 [47] or K27-related ubiquitin enzyme [47,56], may be involved in the process of H2A ubiquitin chain elongation in vivo, which requires further investigation.

### 2.3. Crosstalk of H1 Ubiquitination and H2AK13/15 Ubiquitination

**Figure 6:**
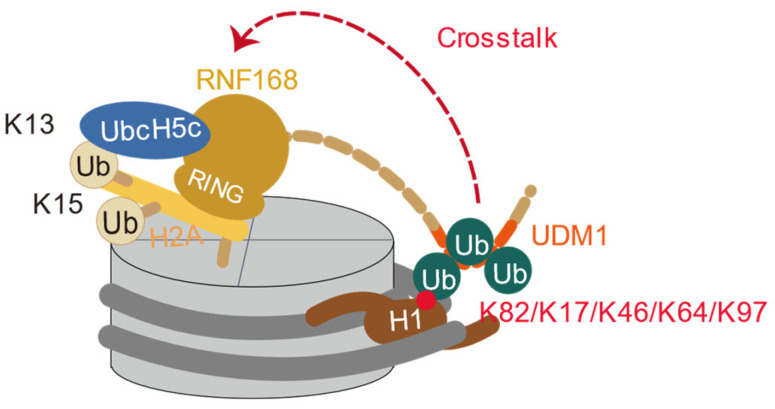
Crosstalk between H1 ubiquitination and H2AK13/15 ubiquitination. The K63-linked polyubiquitinated H1 recruits the UDM1 domain of RNF168 to facilitate the H2AK13/15 ubiquitination by RNF168 RING domain and UbcH5c.

During DNA double-strand break repair, E3 ligase RNF8 is recruited to DSB sites through the Mediator of DNA damage Checkpoint 1 (MDC1) and γH2AX-dependent pathways [59]. RNF8 cooperates with E2 conjugating enzyme UBC13 to catalyze K63-linked polyubiquitin chains at linker histone H1, leading to a looser binding of H1 and chromatin binding [34]. Although DSBs neither affect overall nuclear H1 mobility nor alter H1 subtype distribution at DSB sites, K63-polyubiquitinated H1 is specifically recognized by the UDM1 domain of RNF168 [60]. In vitro experiments have explored how H1 ubiquitination regulates RNF168 recruitment (Figure 6) [60,61]. In vitro ubiquitination assays using recombinant purified full-length RNF168 (RNF168^FL^) and its truncated versions (RNF168^1−193^, RNF168^1−159^, RNF168^1−113^) demonstrated that K63-linked tri-ubiquitinated H1.0 stimulated RNF168 ubiquitination activity similarly to RNF168^FL^, while the shorter RNF168^1−159^ and RNF168^1−113^ constructs were inactive [60], confirming that the RING and UDM1 domains in RNF168^1−193^ form the minimal functional unit [60]. Additionally, K63-linked di-ubiquitin chains are the minimum requirement for activation of RNF168, with the ubiquitin chain length positively correlated with the activation efficiency [60]. Apart from the RNF168 activity stimulated by the different ubiquitin chain lengths on H1.0, the different ubiquitinated sites on H1 were also investigated by the Fierz group, which revealed that di-ubiquitination of histone H1 at different positions (K17, K46, K64, K97) activates RNF168 in a position-dependent manner, with the N-terminal flexible H1K17Ub2 (di-ubiquitination at lysine 17) showing the strongest activation effect [61]. It was also demonstrated that K63-linked di-ubiquitin modification of H1 acts as a cofactor for RNF168 recruitment, effectively localizing it to DNA damage sites [61]. Cryo-EM analysis revealed that K63-linked tri-ubiquitinated H1.0 enables RNF168 to adopt a structurally flexible configuration, positioning its UDM1 domain to interact with the ubiquitin chain on H1.0 [60], which aligns the H1.0 K82 site on the same side of the nucleosome disc as the RNF168/UbcH5c ubiquitination module and suggests a spatial directionality that facilitates efficient recruitment and ubiquitylation of H2A K13/15 across nucleosomes [60].

## 3. Reading: Effector Proteins of H2AK13/15ub

### 3.1. 53BP1

**Figure 7:**
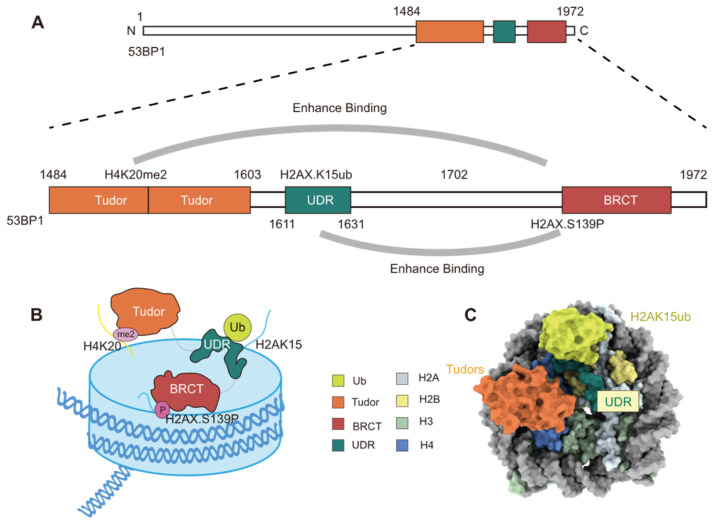
Schematic Diagram of the Binding Mechanism of 53BP1 Protein to Modified Nucleosomes. (**A**,**B**) Cartoon depiction of 53BP1(1484−1972) recognizing the γH2AXK15ub-H4K20me2-modified nucleosome. (**C**) Cryo-EM structure of the 53BP1^TUB^-bound complex formed with the γH2AXK15ub-H4K20me2 nucleosome.

Through yeast two-hybrid screening, 53BP1 was first identified in 1994 as a protein that interacts with the DNA-binding domain of p53 [62]. A subsequent study by Schultz et al. in 2000 demonstrated that 53BP1 plays a pivotal role in the cellular response to DSBs [63]. Following DNA damage, 53BP1 is rapidly phosphorylated by ATM and relocalizes to nuclear foci [64,65]. The role of 53BP1 in DNA damage repair is dependent on its recognition of specific histone modifications: 53BP1 detects the di-methylation of lysine 20 on histone H4 (H4K20me2) via the Tudor domain and H2AK15ub through its ubiquitin-dependent recruitment motif (UDR) (Figure 7A–C) [21]. Further studies have shown that 53BP1 not only specifically recognizes H2AK15 mono-ubiquitination but can also identify di-ubiquitination modifications on H2A (specifically H2AK13diUb and H2AK15diUb), demonstrating its flexibility across different ubiquitination states [66,67]. H4K20me2 is more critical for binding affinity, as the absence of H4K20me2 prevents the binding of 53BP1 to either H2AK13diUb or H2AK15diUb [66,67]. Additionally, the recruitment of 53BP1 is also regulated by other histone modifications, such as H4K16 monomethylation (H4K16me1) [68], H2AX phosphorylation (γH2AX) [69], and secondary ubiquitin modifications (e.g., K6 acetylation [70] and T12 phosphorylation [71]) (Table 1).

#### 3.1.1. Mono-Ubiquitinated H2AK15 Recognition by 53BP1

By recognizing specific histone modifications on chromatin, 53BP1 is recruited to DSB sites during repair. To recognize the H4K20me2, 53BP1 relies on its Tudor domain, and the UDR domain identifies the H2AK15ub, rather than the nearby H2AK13 mono-ubiquitination (Figure 7A) [21]. Wilson et al. employed cryo-EM to resolve, for the first time, the three-dimensional structure of a human 53BP1 fragment in a complex with nucleosomes carrying H4K20me2 and H2AK15ub modifications [73]. Their analysis revealed a unique “sandwich-like” recognition mechanism, where the UDR of 53BP1 passes through the gap between H4 and H2B, extending along the αC helix of H2B and forming a helical structure that precisely positions it on the nucleosome surface [73]. The specific recognition of H2AK15ub by 53BP1, rather than H2AK13ub, is attributed to two arginine residues (Arg11 and Arg17) on the N-terminal tail of H2A [73]. These residues (Arg11 and Arg17) interact with the super-helical DNA, positioning H2AK15ub near the UDR and enhancing the binding affinity of 53BP1 to H2AK15ub [73]. When the ubiquitination shifts to H2AK13, the spatial rearrangement significantly reduces the binding affinity of 53BP1 [73]. The binding mechanism between 53BP1 and histone modifications may provide a foundation for the development of potential drugs aimed at modulating DNA repair pathways.

#### 3.1.2. Di-Ubiquitinated H2AK13/15 Recognition by 53BP1

Studies have reported 53BP1 as a recognition protein specifically for H2AK15monoub, with no affinity for H2AK13monoub [21,73]. Li et al. demonstrated that 53BP1 is also capable of recognizing di-ubiquitination of lysine 13 (Lys13) on H2A [66]. They developed a total chemical synthesis strategy to obtain K27-linked di-ubiquitinated H2A at Lys13 and Lys15 (H2AK13diub and H2AK15diub), which were then used for nucleosome assembly and biochemical studies [66]. Further pull-down assays and immunoblotting showed that 53BP1 binds to both H2AK13diub and H2AK15diub with comparable affinity [66]. In the case of H2AK13diub, the hydrophobic patch centered around the Ile44 residue of the distal ubiquitin is crucial for 53BP1 binding, while the proximal ubiquitin contributes minimally [66]. In contrast, the effective binding of 53BP1 is promoted by both distal and proximal ubiquitin on H2AK15diub [66], demonstrating that H2A Lys13/15 ubiquitination sites provide positional flexibility for 53BP1 recognition [64]. Notably, K48-linked di-ubiquitination at the H2AK13 site also facilitates the recruitment of 53BP1 [67]. Both K27 and K48-linked modifications on H2A exhibit similar binding ability in their interactions with 53BP1, indicating that 53BP1 is highly flexible in recognizing di-ubiquitin chains with different linkages [66,67].

#### 3.1.3. The Second-Tier Modifications of H2AK15ub: K6ac, T12ph

Walser et al. discovered that phosphorylation at Thr12 of ubiquitin occurs at the interface between the UDR motif of 53BP1 and ubiquitin within the 53BP1/NCP-H2AK15ub complex [71]. The phosphorylation at Thr12 specifically disrupts the interaction between 53BP1 and H2AK15ub, while preserving the binding of HR-related factors such as Radiation Sensitive 51 (RAD51) and the BRCA1/BARD1 complex [71]. Disruption of the 53BP1-H2AK15ub interaction selectively suppresses the 53BP1-dependent NHEJ repair pathway, thereby promoting the choice of the HR pathway during DSB repair [71]. Notably, the binding affinity of 53BP1 is also regulated by the acetylation of the H2AK15ub K6 site [70]. Specifically, the EC50 for the interaction between 53BP1 Tudor domain and H2AK15ub-H4K20me2 nucleosome was measured to be 3.1 nM [70]. In contrast, the EC50 for binding between the 53BP1 Tudor domain and a nucleosome containing H2AK15ubK6^Ac^ and H4K20me2 is 13.2 nM, suggesting that Ub-K6 acetylation weakens 53BP1 binding affinity [70]. Structurally, the basic side chain of Ub-K6 is positioned near the acidic residue D1620 in the 53BP1-UDR domain [70]. Acetylation at Ub-K6 potentially blocks the polar charge interactions, thereby weakening the affinity of 53BP1 for the nucleosome [70].

#### 3.1.4. 53BP1 Recruitment Influenced by Other Histone Modifications

H2AX phosphorylation at Ser139, also named γH2X, was reported to directly recruit the 53BP1 during the early stage of DNA damage [69]. In vitro experiments indicate that γH2AX exhibits a binding affinity of 2.7 μM through its interaction with the BRCA1 C-Terminal (BRCT) domain of 53BP1 [69]. The essential binding of 53BP1 to γH2AX through its BRCT domain ensures DNA repair by positioning 53BP1 at damage sites even in the absence of MDC1 [69]. Mutational studies involving the 53BP1 BRCT domain (R1811Q and K1814M) revealed that these mutations significantly impair the ability of 53BP1 to bind γH2AX [69]. Furthermore, the binding of 53BP1 and γH2AX facilitates the rapid accumulation of 53BP1 at DNA damage sites, enhancing repair efficiency, particularly in the repair of heterochromatin and telomeres [69]. Interestingly, when H2AXK15 ubiquitination and H4K20 di-methylation are also present, the phosphorylation of H2AX at Ser139 alters the interface between ubiquitin and 53BP1, which disrupts the expected synergistic effects of the three marks, instead demonstrating functional redundancy [72].

Additionally, Lu et al. reported that H4K16 monomethylation (H4K16me1), catalyzed by the histone methyltransferase G9a-like protein (GLP), rapidly increases in response to DNA damage [68]. The H4K16me1 enhances the ability of 53BP1 to bind damaged chromatin [68]. Unlike the acetylation of H4K16, H4K16me1 directly interacts with the Tudor domain of 53BP1 and cooperates with H4K20me2 to enhance 53BP1 recruitment and NHEJ repair efficiency [68]. Recent studies have highlighted the synergistic role of H4K16me1 and H4K20me2 in promoting 53BP1 recruitment [68]; however, the specific molecular conformation still needs to be elucidated through higher-resolution structural biology techniques, such as cryo-EM or X-ray crystallography. In particular, cryo-EM and X-ray crystallography could clarify the interaction interface between the 53BP1 Tudor domain and the dual histone marks (i.e., H4K16me1 and H4K20me2), providing insights into the mechanisms that drive the synergy between these histone modifications in promoting 53BP1 recruitment.

### 3.2. RAD18

Early research indicated that the yeast RAD18 participates in post-replication repair (PRR), a role later confirmed for its human homolog, RAD18 [74,75]. RAD18, an E3 ubiquitin ligase encoded by the human *RAD18* gene, regulates DSB repair by binding to H2A ubiquitinated by RNF168 [75,76]. Stimulated emission depletion (STED) microscopy studies revealed that RAD18 localizes to chromatin nano-regions within the DNA damage repair center, limiting 53BP1 to peripheral areas and thereby effectively inhibiting NHEJ [75]. Nuclear magnetic resonance (NMR) studies identified that key amino acid residues in RAD18 (positions 198–240) are responsible for its binding to H2A ubiquitinated at K15 [38]. Specifically, the RAD18 basic residue R234 plays a crucial role in interacting with nucleosomes [38]. Its UBZ (Ubiquitin-Binding Zinc finger) domain occupies a unique position between ubiquitin and the H2A-H2B surface, with its C-terminal extension interacting with the H2A-H2B acidic patch [38]. However, the autoubiquitination of RAD18 at lysine residues K186, K197, K201, and K218 impairs its binding to ubiquitinated chromatin by making its UBZ domain inaccessible, thus reducing its dwell time at damage sites [75]. Additionally, mutations in the UBZ domain of RAD18 (e.g., H219Y and R226C) lead to significant functional defects in UV-induced damage foci, where they fail to efficiently bind ubiquitinated H2A and K63-linked ubiquitin chains [75]. Deficiencies in translation DNA synthesis, DNA gap filling, and DSB repair, resulting from the loss of RAD18 function, may drive genomic instability in cancer cells [75].

### 3.3. BARD1

#### 3.3.1. Discovery and Functionality of BARD1

**Figure 8:**
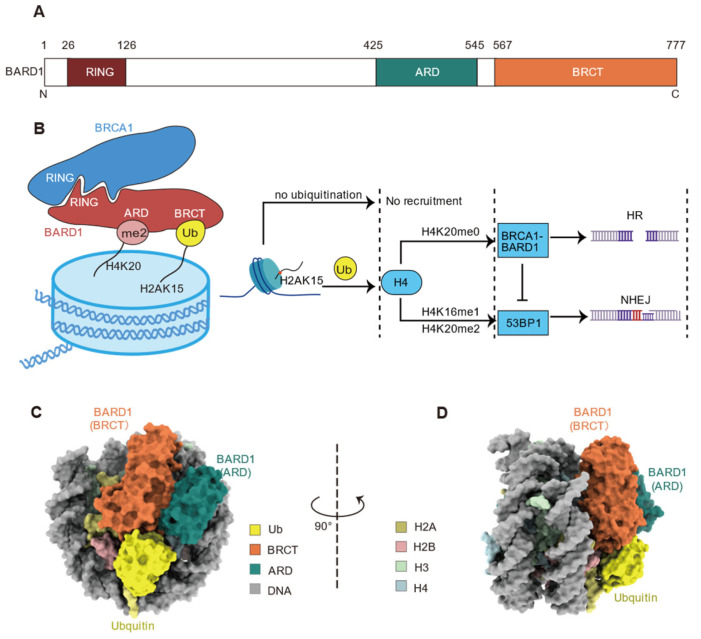
The BARD1 domain composition, the BRCA1-BARD1 complex in DNA repair pathway regulation, and the structure of BARD1 and H2AK15ub nucleosome complex. (**A**) Schematic representation of BARD1 domains, highlighting the RING domain (residues 26–126), ankyrin repeat domain (ARD, residues 425–545), and BRCT domain (residues 567–777). (**B**) A proposed model further illustrates the bivalent recognition of nucleosomes by BARD1, with a logic gate mechanism that highlights how the interplay of H2AK15ub and H4K20 post-translational modification states govern the decision between HR and NHEJ. (**C**,**D**) Cryo-EM reconstruction (left and right) of the BARD1 complexed with the H2AK13/15ub nucleosome is shown in two orientations, emphasizing the interaction interfaces between the BARD1 (BRCT and ARD domains) and the ubiquitin and nucleosome components (H2A, H2B, H3, H4, and DNA).

BARD1 (BRCA1-associated RING domain protein 1) was initially identified in 1996 and is known to interact with BRCA1 [77]. The resulting BRCA1/BARD1 complex accurately repairs DNA through the HR pathway during DSB repair [77]. BRCA1 and BARD1 form a heterodimer via their respective N-terminal RING domains, endowing the complex with E3 ubiquitin ligase activity for the ubiquitination at the C-terminal of H2A (K125/127/129) (Figure 8A,B) [77,78]. Recent studies have further elucidated that the BRCA1/BARD1 complex, through the BRCT domain of BARD1, selectively recognizes RNF168-mediated ubiquitination modifications at H2A K13 and K15 sites [22,78,79,80]. Additionally, the ankyrin repeat domain (ARD) domain of BARD1 exhibits a preferential binding affinity for unmethylated H4K20 (H4K20me0), but not the methylated one [22,80]. The binding of the ARD domain to unmethylated H4K20 potentiates the high-affinity association between the complex and damage sites, which not only ensures the proper localization of the BRCA1-BARD1 complex to post-replicative chromatin regions but also promotes HR while inhibiting the alternative NHEJ pathway.

In the choice of DNA repair pathway, BRCA1/BARD1 competes with 53BP1. Both BARD1 and 53BP1 bind to H2AK15ub, but 53BP1 specifically recognizes H4K20me2, while BARD1 preferentially binds to H4K20me0 (Figure 8B) [22]. The differential recognition of H4K20me2 by 53BP1 and H4K20me0 by BARD1 play a critical role in determining the repair pathway, with 53BP1 promoting the error-prone NHEJ pathway, whereas BRCA1/BARD1 favors the high-fidelity HR pathway [22]. Defects in the BRCA1-BARD1 complex result in reduced HR repair efficiency, leading cells to preferentially engage the error-prone NHEJ pathway, which in turn increases cancer susceptibility [22]. The proper function of BRCA1-BARD1 is particularly critical in cancers associated with BRCA1 mutations, such as breast and ovarian cancers [22,78,79].

#### 3.3.2. Mechanism of BARD1 Recognition of H2AK13/15ub

Recently, the Mer group and Zhou group independently reported the cryo-EM structures of BARD1 BRCT and ARD domains in complex with H2AK13/15 ubiquitinated nucleosomes (Figure 8C,D) revealing three major interacting surfaces: H2AK13/15ub, H4K20me0, and H2A-H2B acidic patch [78,79,80]. In particular, regarding the recognition of H2AK13/15ub, the BRCT domain of BARD1 specifically recognizes H2AK15ub through direct binding [22,78,79,80]. The D710 and Q715 residues of BARD1 span the T66 site on H2AK15ub, forming multiple hydrogen bonds, and N718 establishes an electrostatic interaction with the N60 residue of H2AK15ub [78,79]. Additionally, the D729 and R751 residues of BARD1 establish salt bridges with the K63 and E64 residues of H2AK15ub, respectively [78,79]. These interactions are further stabilized by electrostatic contact between K754 of BARD1 and the S65 residue of H2AK15ub [78,79].

The ARD domain of BARD1 binds to H4K20me0, with the ε-amino group of H4 K20 forming salt bridges with the E429, D458, and E467 residues of BARD1 [78,79]. The tail of the histone H4 extends along the surface of the ARD domain of BARD1, passing through an “acidic pocket” formed by the carboxylate groups of E429, D458, and E467 in ARD before reaching the folded structure [78]. The H18 residue of H4 sits within the binding pocket of BARD1, while the unmethylated K20 inserts into the acidic channel of the ARD, forming hydrogen bonds with the E429, D458, and E467 residues of BARD1 [78]. Mutating BARD1 residues E467, N470, and D500 to alanine significantly weakens the affinity of BARD1 for ubiquitinated nucleosomes and reduces the accumulation of BRCA1-BARD1 complex at DNA double-strand break sites [78]. Additionally, the R427 residue of BARD1 points toward the DNA phosphate backbone, forming additional electrostatic interactions that further enhance the stability of the ARD domain to H4K20me0 [78].

The H2A-H2B acidic patch, an interacting hot spot of chromatin effector proteins (e.g., Dot1L [81,82,83,84,85,86], RNF168 [19,20,39], SSX1 [87]), is also engaged by the BARD1 [78,79]. The R705 residue in BARD1 forms a charge interaction network, or “salt bridge”, with the acidic triad of H2A (E61, D90, and E92) [79]. R705 is referred to as the “arginine anchor” residue, as it firmly secures BARD1 to the nucleosome. Another BARD1 residue, K708, further stabilizes the binding through charge interaction with E64 on H2A [79].

Interestingly, the recognition of H2AK13/15 ubiquitination by BARD1 facilitates the inherent ubiquitin E3 ligase activity of the BRCA1/BARD1 complex, enabling the ubiquitination of the nucleosomal H2A C-terminus at residues K125/127/129 [78], which displaces 53BP1-mediated resection barriers and enables extended DNA end processing [88]. Considering the long-flexible linkage between the BARD1 ARD-BRCT domain and the N-terminal RING domain (Figure 8A) [78], the recognition of H2AK13/15ub by ARD could theoretically stimulate crosstalk either within a single nucleosome or between multiple nucleosomes [80]. However, this potential crosstalk process remains to be further explored through biochemical and structural investigations.

## 4. Erasing: H2AK13/15 Deubiquitinases

**Figure 9:**
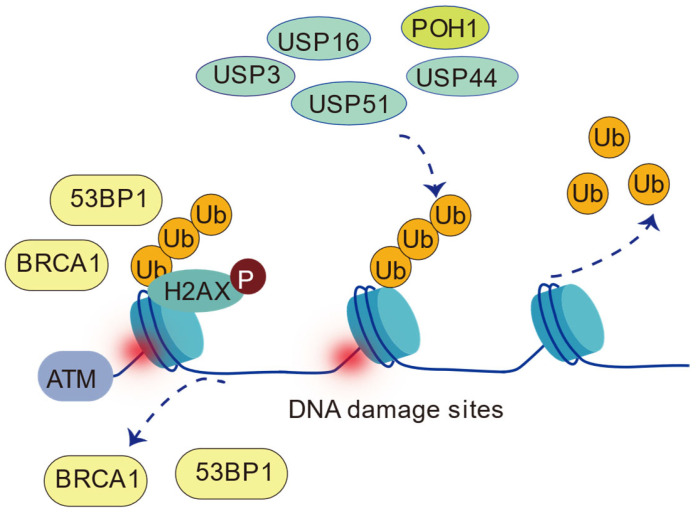
Deubiquitinases (USP16, USP3, USP51, POH1, and USP44) regulate H2AK13/15ub. USP16, USP3, and USP51 directly remove H2AK13/15ub, while USP44 and POH1 indirectly regulate it by affecting upstream ubiquitination events. Note: USP16, USP3, USP51, and USP44 belong to the USP family; POH1 belongs to the JAMM/MPN+ family.

The ubiquitination of H2AK13/15 plays a central role in the cellular response to DNA damage, particularly in the selection of HR or NHEJ repair pathways and the recruitment of repair factors (such as 53BP1, BARD1, RAD18, etc.). Several deubiquitinating enzymes have been reported to precisely regulate the DNA repair process by removing H2AK13/15 ubiquitination modifications, thereby maintaining genomic stability (Figure 9).

### 4.1. USP3

USP3, a newly characterized human ubiquitin-specific protease, was first reported by Sloper-Mould et al. in 1999 as a partial cDNA clone resembling one of two conserved sequence regions typical of all ubiquitin-specific proteases [89]. In 2007, Nicassio et al. demonstrated that USP3 deficiency significantly increases the levels of monoubiquitinated H2A (uH2A) and γ-H2AX, resulting in abnormalities in DNA replication and recombination, as well as the accumulation of DNA breaks [90]. Elevated levels of uH2A and γ-H2AX further activate ATR/ATM-dependent checkpoint responses, exacerbating genomic instability [90]. USP3 regulates DNA damage repair signaling by deubiquitinating the K13 and K15 sites on H2A and H2AX, thereby reversing ubiquitination modifications mediated by RNF168 [26]. During DNA damage repair, USP3 negatively regulates the accumulation of BRCA1 and 53BP1 at DNA break sites by deubiquitinating Ub-conjugates of γH2AX (Ub-γH2AX) and uH2A [26]. Additionally, USP3 modulates the duration of the phosphorylation on these proteins during DNA damage repair [26]. Moreover, USP3 influences chromatin structure at the damage sites, preventing the excessive accumulation of DNA repair factors and ensuring the appropriate selection and regulation of the repair pathways [26,90]. The involvement of USP3 in the regulation of DNA damage repair warrants further investigation of its functional aspects in DNA break repair, cell cycle and survival, and carcinogenesis.

### 4.2. USP16

USP16, also known as Ubiquitin-Processing Protease M (Ubp-M), serves as a negative regulator of ubiquitin foci induced by DNA damage and the damaged DNA repair process [27,91]. ChIP-seq studies have shown that USP16 predominantly localizes to gene promoter regions. Knockout of the *USP16* gene results in the accumulation of H2Aub at these sites, leading to enhanced gene repression [91]. USP16 was first identified in 2007 as the primary deubiquitinase for H2AK119ub, exhibiting catalytic activity on nucleosome substrates rather than octamer substrates [92]. Recent studies have elucidated the structural mechanism by which the monomeric deubiquitinase USP16 removes H2AK119ub, revealing a novel nucleosomal deubiquitination recognition mode that does not rely on the H2A-H2B acidic patch [93]. In addition, the relationship between USP16 and DNA damage repair is primarily reflected in its ability to reverse RNF168-mediated H2AK13/15 ubiquitination, where USP16 deubiquitinates H2A in response to DNA damage [27]. Furthermore, HERC2 interacts with the disordered region of USP16 (residues 136–185) through its C-terminal HECT domain (residues 4421–4834), increasing the intracellular expression of USP16 [27]. Knockdown of HERC2 leads to reduced USP16 protein levels, followed by an accumulation of H2A ubiquitination [27]. However, whether HERC2 directly regulates the deubiquitinase activity of USP16 in vitro remains to be further explored.

### 4.3. USP44

USP44 was reported in 2007 for its role in the spindle assembly checkpoint [94] and was later found to play a critical role in DSB repair [28]. Knockdown experiments show that USP44 antagonizes ubiquitin chain formation on H2A, regulating the ubiquitin-dependent DSB repair response to prevent excessive recruitment of repair proteins and ensuring the balance and efficiency of the DNA damage repair [28]. Specifically, when H2A and ubiquitin were ectopically co-expressed, wild-type USP44 was shown to reduce the levels of mono-, di-, and tri-ubiquitylated H2A [28]. In contrast, overexpression of the inactive USP44 (C282A) mutant significantly increased the formation of higher-order ubiquitin chains on H2A and hindered the formation of RAP80 foci, which recruit the BRCA1-A complex to DSB sites [28], indicating that USP44 regulates the ubiquitin-dependent DSB repair pathway by inhibiting ubiquitin chain formation on H2A [28]. Future research is still needed to further verify whether USP44 can directly mediate the deubiquitination of specific ubiquitination sites on H2A, such as H2AK119 or H2AK13/15, in vitro.

### 4.4. USP51

The role of USP51 in the deubiquitination of H2AK13/15ub was identified by Wang et al. in 2016 [29]. USP51 directly interacts with the H2A-H2B dimer and modulates H2AK15ub accumulation, promoting the assembly of repair proteins during the initial phase of DNA damage [29]. Upon completion of the repair process, the deubiquitinating activity of USP51 facilitates the disassembly of repair proteins and the restoration of chromatin to its original state [29]. In vitro studies have shown that USP51 can efficiently remove H2AK13/15ub and H2AK119ub, along with H2BK120ub, but not H2BK34ub [95]. The depletion of USP51 not only leads to delayed disassembly of proteins at DNA damage sites but also significantly affects cell survival and repair capacity, indicating that USP51 plays a crucial role in maintaining the dynamic balance of the DNA damage repair and genomic integrity through the regulation of H2AK13/15ub [29]. Further investigation into the specific mechanisms of USP51 in DNA damage repair and its potential additional substrates will offer new insights into understanding DNA damage repair and developing related therapeutic strategies.

### 4.5. POH1

The proteasomal deubiquitinating enzyme POH1 is crucial for regulating ubiquitin conjugates generated in response to DNA damage [30,31]. Although POH1 does not directly remove the H2AK13/K15ub modification, it counteracts K63-linked polyubiquitin chains mediated by RNF8/RNF168, preserves the presence of jumonji domain-containing protein 2A (JMJD2A) on chromatin, limits the excessive accumulation of 53BP1 at sites of DNA damage, and helps to balance the repair signals mediated by H2AK13/15ub [31]. In the absence of POH1, K63 ubiquitin chains accumulate at DNA damage sites, resulting in an overextension of repair signals that disrupt the normal repair process reliant on H2AK13/15ub [31]. Furthermore, POH1 facilitates RAD51 loading and participates in HR repair independently of 53BP1 [31]. Cells deficient in POH1 demonstrate increased sensitivity to DNA-damaging agents, highlighting its essential role in sustaining the DNA damage response [31]. By regulating the removal of ubiquitin chains, POH1 not only balances the accumulation of repair proteins and the propagation of ubiquitination signals but also indirectly modulates the dynamics of H2AK13/15 ubiquitination, thereby ensuring the accuracy and efficiency of DNA repair [31].

## 5. H2AK13/15 Ubiquitination and Disease

**Figure 10:**
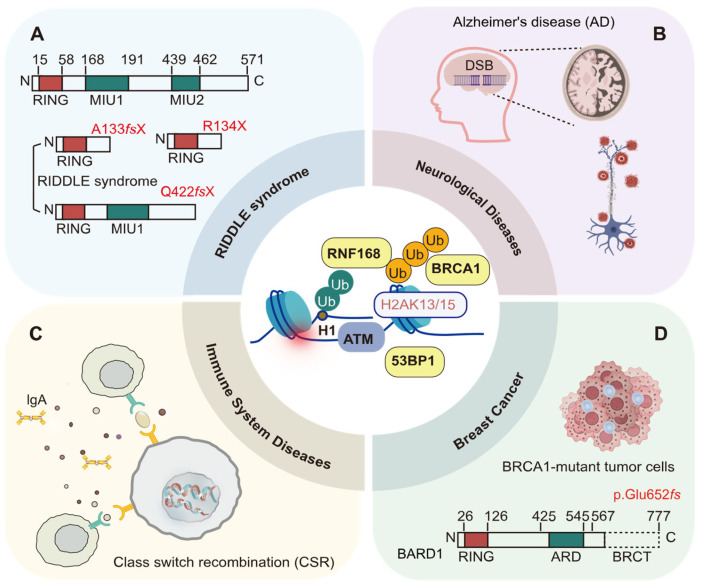
Disease associations of H2AK13/15 ubiquitination. (**A**) Mutations in the RNF168 gene (e.g., A133fsX and Q442fsX) disrupt H2AK13/15 ubiquitination. (**B**) The BRCA1/BARD1 complex, 53BP1 repair factors showed abnormal localization and abundance, failing to be effectively recruited by H2AK13/15ub to DSB sites. Defective H2AK13/15 ubiquitination reduces the efficiency of DNA damage repair in neurons. (**C**) RNF168 deficiency significantly reduces CSR efficiency, declining the efficiency of CSR to IgA and weakening immune responses. (**D**) BRCA1 promotes HR repair by interacting with H2AK13/15 ubiquitination. BRCA1 mutations impair HR repair, resulting in genomic instability. Concurrent BRCA1 and BARD1 mutations (e.g., p.Glu652fs) synergistically disrupt DNA repair mechanisms, leading to significantly increased breast cancer risk. Figure created with BioRender.com.

H2AK13/15 ubiquitination plays a pivotal and dynamic role in the DNA damage repair process through its “writer”, “reader”, and “eraser” mechanisms. Disruption of any of these processes results in genomic instability and cellular dysfunction. RNF168, as the “writer”, specifically catalyzes ubiquitylation at histone H2A K13 and K15 residues. Loss of RNF168 function severely compromises the DNA damage response by disrupting H2AK13/15 ubiquitination [10]. Abnormalities in the “reading” of the H2AK13/15 ubiquitination mark hinder the proper localization of repair factors such as 53BP1 and BRCA1 [21,22], which leads to delayed or incomplete repair and exacerbates DNA damage accumulation and genomic instability. The resulting defects in DNA repair mechanisms have been implicated in the development of various diseases, playing a critical role in conditions such as RIDDLE syndrome [96], neurodegenerative disorders [23], immune system dysfunctions [24,97], and breast cancer (Figure 10) [25].

### 5.1. RIDDLE Syndrome

In 2007, Stewart et al. reported that RIDDLE syndrome (radiosensitivity, immunodeficiency, dysmorphic features, and learning difficulties), a rare genetic immunodeficiency disorder, is caused by biallelic mutations in the RNF168 gene [96]. The first RNF168 mutation is a duplication of a guanine (G) nucleotide at position 397 (c.397dupG), which causes a frameshift and produces a truncated protein, p.Ala133GlyfsX11 (Figure 10A) [96]. The mutation replaces alanine (Ala) at position 133 with glycine (Gly), followed by 12 additional amino acids from the shifted reading frame, ultimately introducing a stop codon at position 11 of the new frame (A133fsX) [96]. The second mutation is a deletion of nucleotides 1323–1326 (c.1323_1326delACAA), also resulting in a frameshift that generates a truncated protein, p.Gln442LysfsX45. Here, glutamine (Gln) at position 442 is replaced by lysine (Lys), followed by 46 additional amino acids from the shifted frame, culminating in a stop codon at position 45 (Q442fsX) [47]. Both mutations preserve the E3 ubiquitin ligase catalytic domain (the RING finger) of the RNF168 protein but result in the loss of the ubiquitin-interacting motif (MIU) domains [47]. Specifically, the A133fsX mutant lacks both MIU domains—UDM1 (residues 9–40) and UDM2 (residues 146–170)—along with the intervening residues, resulting in a complete disruption of ubiquitin-binding and regulatory pathways essential for DNA double-strand breaks repair. In contrast, the Q442fsX mutant retains the UDM1 domain (residues 9–40) but loses UDM2 (residues 146–170), partially impairing its function in the DNA damage response. [47]. The functional loss of RNF168 caused by these mutations impairs the ubiquitination of histone H2A at K13/15, which in turn disrupts the recruitment of critical DNA damage response factors, such as 53BP1 and BRCA1, to DSB sites [96]. Dysfunctional RNF168 results in persistent ATM-dependent DNA damage signaling, characterized by the abnormal accumulation of γ-H2AX, MDC1, and Nbs1 foci, excessive phosphorylation of these proteins, and delays in the G2/M cell cycle checkpoint [47,59,96,98,99].

### 5.2. Neurological Diseases

In the nervous system, neurons are terminally differentiated cells, and impairments in DNA double-strand break repair functions directly affect neural function [23]. Suberbielle et al. found that in the brains of Alzheimer’s disease (AD) patients, the levels of BRCA1 were significantly reduced, while the levels of other DNA repair factors, such as MRE11, NBS1, and RAD51, remained unchanged (Figure 10B) [23]. The BRCA1/BARD1 complex, RAP80, and RAD51 repair factors showed abnormal localization and abundance, failing to be effectively recruited by H2AK13/15ub to DSB sites, which impaired DNA damage repair efficiency [23]. Further investigations using mouse models revealed that physiological neuronal activity can induce a temporary increase in DSBs in neurons, which are typically repaired swiftly under normal conditions [23]. However, in AD transgenic mice, reduced BRCA1 levels impair DNA repair, preventing timely repair of these DSBs and leading to their persistence [23]. Additionally, specific knockdown of BRCA1 expression in the dentate gyrus resulted in increased neuronal DSBs, neuronal atrophy, impaired synaptic plasticity, and cognitive deficits without triggering apoptosis [23]. Future studies should explore whether BRCA1 participates in neuronal DNA repair through the NHEJ pathway and whether it has other unique functions in neurons beyond DNA repair. Therefore, enhancing DNA repair mechanisms may offer a promising therapeutic strategy for addressing neurodegenerative diseases, such as Alzheimer’s.

### 5.3. Immune System Diseases

According to the study by Santos et al. in 2010, the ubiquitination of H2AK13/15 sites promotes DNA damage repair and class switch recombination (CSR), maintaining genomic stability and supporting the normal function of the immune system (Figure 10C) [24]. In mice lacking RNF8, there is a significant reduction in CSR efficiency, along with the accumulation of unrepaired DSBs around the immunoglobulin heavy chain (IgH) locus [24]. The accumulation of DNA damage leads to genomic instability, ultimately impairing B-cell function and antibody production [24]. Additionally, in CF12F3-3 cells where RNF8 and RNF168 expression was reduced using shRNA, there was a notable decline in the efficiency of CSR to IgA [97]. Interestingly, 53BP1 regulates immune cell survival through its direct interaction with p53, independently of the RNF8 and RNF168 pathways [97]. Future investigations are necessary to further elucidate how RNF8 and RNF168-mediated H2AK13/15 ubiquitination affects DNA damage repair and CSR, and to clarify the dependencies and independencies of the 53BP1 pathway in immune regulation. Additionally, these findings may have important implications for the diagnosis and treatment of immune-related diseases.

### 5.4. Breast Cancer

Breast cancer is the cancer with the highest incidence and mortality rates among women, a trend that remains on the rise [100,101]. Research has shown that hereditary factors are involved in the occurrence and progression of breast cancer, with BRCA1/2 gene mutations linked to familial breast cancer cases (Figure 10D) [102]. Additionally, the BRCA1 protein interacts with the RNF168 to regulate the ubiquitination of H2AK13/15 sites, participating in the HR repair of DNA double-strand breaks [103]. Pathogenic mutations in BRCA1/2 genes impair DNA damage repair mechanisms, resulting in genomic instability and significantly increasing the risk of breast cancer, particularly in mutation carriers who develop the disease before the age of 70 [103]. De Brakeleer et al. conducted a study screening 196 high-risk breast cancer families, where they reported a protein-truncating mutation in the BARD1 gene (p.Glu652fs) [102]. The p.Glu652fs mutation in the BARD1 gene leads to the loss of the functional BRCT domain of the BARD1 protein, highlighting the association between BARD1 mutations and an increased risk of breast cancer [103]. Additionally, the RNF168 level is higher in breast cancer tissues than in normal breast tissues [41]. The estrogen receptor α (ERα), a key player in breast cancer development, is influenced by RNF168, which interacts with the promoter region of ERα to enhance its transcriptional activity, thereby boosting the ERα signaling cascade and promoting the proliferation of ERα-positive breast cancer cells [41]. However, the loss of RNF168 in BRCA1/2-mutated breast cancer cells has also been reported to lead to R-loop accumulation, which triggers double-strand breaks, senescence, and eventual cell death [25]. The specific role of RNF168 in BRCA1-mutant tumor cells warrants further investigation, especially regarding its protein levels and how they influence oncogenic function.

## 6. Conclusions and Discussion

Histone H2A ubiquitination at K13/15 represents an emerging important epigenetic modification system that plays a crucial role in DNA damage repair. In this article, we provide an in-depth overview of the molecular mechanisms that regulate the writing, reading, and erasure of H2A K13/15 ubiquitination, along with a discussion of its implications for disease development. In light of the current understanding of the H2AK13/15 ubiquitination code system, we foresee several critical areas that merit further investigation. Firstly, the structural basis of the RNF168-mediated H2AK13/15 homotypic ubiquitin chain formation, particularly K27- and K63-linked chains, needs to be elucidated. This includes identifying the key regulatory factors that facilitate chain extension. For example, the classical E2-E3 pair UBC13 and TRAF6 demonstrate distinct roles in K63 chain formation [40], with UBC13 catalyzing chain extension and TRAF6 providing substrate specificity, which plays a pivotal role in DNA damage response. Secondly, the redundancy in the dual-site ubiquitination of H2A at K13 and K15 likely serves as an additional “backup” mechanism to ensure the fidelity of the DNA damage repair response, particularly in the presence of H2AK13 or K15 mutations [19,39]. Moreover, secondary modifications of Ub play crucial roles in regulating ubiquitination signals. For instance, the second-tier modifications of H2AK15ub, such as T12 phosphorylation and K6 acetylation, attenuate the binding of effector proteins [70,71]. It could be valuable for future studies to investigate further key secondary modification sites, their regulatory mechanisms, and possible interactions or synergistic effects among these modifications.

The specific antibody targeting H2AK13/15ub plays a crucial role in enabling in vivo imaging and mapping studies, as well as facilitating in vitro tests for ubiquitination activity. Wang et al. developed a highly specific H2AK15ub antibody, which exhibits high specificity for both mono- and di-ubiquitinated H2A catalyzed by RNF168 but does not recognize unmodified H2A [29]. The antigen design used a branched H2AK15 peptide (Ac-ARAKAK[GGRL]TRSSC) conjugated with the keyhole limpet hemocyanin (KLH) carrier protein [29]. Additionally, the commercially available H2AK15ub antibody (Merck/Sigma: MABE1119, St. Louis, MO, USA) has proven to be effective for applications such as Western blotting and immunofluorescence, making it a valuable tool for researchers studying ubiquitination processes. However, specific antibodies for H2AK13ub remain scarce. Future efforts may focus on (1) developing antibodies that specifically distinguish between H2AK13 and H2AK15 ubiquitination to clarify their distinct roles in the DNA damage response; (2) developing antibodies that can recognize dual-site ubiquitination (i.e., H2AK13ub-K15ub) to explore the cooperative functions of the modifications in repair pathway selection; and (3) optimizing antibody performance for nucleosome conformation-dependent applications like ChIP-seq and immunohistochemistry. In these techniques, the interaction between antibodies and target proteins depends not only on the peptide sequence but, more critically, on the spatial conformation of the proteins. This conformational specificity requires antibodies capable of recognizing and binding to distinct three-dimensional structures of target proteins. Improving antibody performance in such conformation-sensitive assays will enhance the reliability and reproducibility of results. These advancements will deepen our understanding of the H2AK13/15 ubiquitination system in DNA damage repair and may open new possibilities for disease diagnosis and the development of therapeutic strategies.

## Figures and Tables

**Table 1:** Domains of 53BP1 protein-recognized histone modifications and their impact on binding affinity.

53BP1 Domain	Histone Modification	Influence	Refs.
Tudor	H4K20me2 H4K16me1	Enhance binding	[21,68]
UDR	H2AK15monoub H2AK15diub H2AK13diub	Enhance binding	[21,66,67]
	H2AK15Ub^T12ph^ H2AK15Ub^K6ac^	Impair binding	[70,71]
BRCT	γH2AXS139	Enhance binding	[69,72]

## Data Availability

Not applicable.
